# Uptake of the human papillomavirus vaccine in Kenya: testing the health belief model through pathway modeling on cohort data

**DOI:** 10.1186/s12992-016-0211-7

**Published:** 2016-11-15

**Authors:** Heleen Vermandere, Marie-Anne van Stam, Violet Naanyu, Kristien Michielsen, Olivier Degomme, Frans Oort

**Affiliations:** 1 International Centre for Reproductive Health, Ghent University, De Pintelaan 185, ingang 75, UZP 114, 9000 Ghent, Belgium; 2 University Medical Centre Utrecht, Utrecht, The Netherlands; 3 Department of Behavioral Sciences, School of Medicine, College of Health Sciences, Moi University, Eldoret, Kenya; 4 Department of Child Development and Education, University of Amsterdam, Amsterdam, The Netherlands

**Keywords:** HPV vaccination, Health Belief Model, Cohort, Kenya, Pathway modeling

## Abstract

**Background:**

Many studies investigate HPV vaccine acceptability, applying health behavior theories to identify determinants; few include real uptake, the final variable of interest. This study investigated the utility of the Health Belief Model (HBM) in predicting HPV vaccine uptake in Kenya, focusing on the importance of promotion, probing willingness to vaccinate as precursor of uptake and exploring the added value of personal characteristics.

**Methods:**

Longitudinal data were collected before and after a pilot HPV vaccination program in Eldoret among mothers of eligible girls (*N* = 255). Through pathway modeling, associations between vaccine uptake and the HBM constructs, willingness to vaccinate and adequate promotion were examined. Adequate promotion was defined as a personal evaluation of promotional information received. Finally, baseline cervical cancer awareness and socio-demographic variables were added to the model verifying their direct, mediating or moderating effects on the predictive value of the HBM.

**Results:**

Perceiving yourself as adequately informed at follow-up was the strongest determinant of vaccine uptake. HBM constructs (susceptibility, self-efficacy and foreseeing father’s refusal as barrier) only influenced willingness to vaccinate, which was not correlated with vaccination. Baseline awareness of cervical cancer predicted uptake.

**Conclusions:**

The association between adequate promotion and vaccination reveals the importance of triggers beyond personal control. Adoption of new health behaviors might be more determined by organizational variables, such as promotion, than by prior personal beliefs. Assessing users’ and non-users’ perspectives during and after implementing a vaccination program can help identifying stronger determinants of vaccination behavior.

**Electronic supplementary material:**

The online version of this article (doi:10.1186/s12992-016-0211-7) contains supplementary material, which is available to authorized users.

## Background

Cervical cancer poses a high burden on women’s health in Kenya due to its high incidence and the poor prognosis of most patients. This elevated incidence rate is related to the high prevalence of HIV, the low screening coverage in Kenya (only 3.2 % of all women are screened every 3 years), and the absence of the Human Papillomavirus (HPV) vaccine in the national vaccination program [1]. If the HPV vaccine becomes available in Kenya, it would provide women on-going protection against several high-risk HPV types [2–4].

However, before adding the HPV vaccine to a national vaccination program, a situation analysis is valuable to prepare the introduction of the vaccine in terms of costs and infrastructure but also to assess readiness among the population [5, 6]. Worldwide, many studies have investigated girls’ caregivers’ willingness to vaccinate, often before the vaccine was introduced. While acceptability is usually high, doubts about the safety and efficacy of the vaccine are common [7–11]. In certain subpopulations, there is also the belief that the vaccines might promote promiscuity although past research does not support these claims [12, 13].

Frequently, these acceptability studies apply (health) behavior theories that include a variety of factors (e.g. attitudes, beliefs, perceived barriers) which are believed to influence the likelihood of a certain action [14, 15]. By investigating these theories’ constructs, researchers aim to identify determinants of vaccine uptake and refusal to incorporate them in vaccination strategies. An example of such theory is the ‘Health Belief Model’ (HBM), an established model often used to identify determinants of vaccination behavior [14, 16]. The original HBM indicates that in order for an individual to take action (e.g. to vaccinate your daughter), this person would have to (1) perceive the disease at least as ‘moderately severe’; (2) perceive a susceptibility or vulnerability to the disease; (3) believe that there are benefits in taking the preventive action; and (4) not perceive major barriers obstructing the action. According to the theory, the likelihood to action increases when the perceived benefits outweigh the perceived barriers [17]. Additionally, the HBM is often extended with two more constructs: (5) self-efficacy, indicating the *‘expectancies about one’s own competence to perform the behavior’* and (6) cues to action (CTA), i.e. *‘the specific stimuli necessary to trigger the decision-making process’* [18–20].

Brewer et al. (2007) and Cunningham et al. (2014) have reviewed HPV vaccine acceptability studies focusing on the HBM constructs in the USA and Africa respectively [14, 21]. The former review included twenty eight studies, the latter fourteen (among ten countries). Perceived susceptibility reported in African studies was not always high which might have been caused by misunderstandings such as believing the disease is inherited. In general, own risk was considered lower than a daughter’s risk of HPV infection or cervical cancer. While studies in the USA revealed a positive relation between susceptibility and acceptability [14] Cunningham et al. (2014) reported either no correlation [22] or also a positive one [21, 23]. Among all studies, the majority of the participants agreed that cervical cancer is a serious illness (perceived severity) [14, 21]. While two studies, in Botswana and Ghana [22, 23], detected an association between HPV vaccine acceptability and perceived severity, the other studies were not conclusive. Perceived effectiveness of the HPV vaccine was the main benefit investigated while in terms of barriers cost and safety concerns were discussed, among others. The link with acceptability remains again unclear for both constructs: reported barriers do not necessarily deter acceptability and trusting the vaccine’s efficacy does not always lead to higher willingness to vaccinate [14, 21]. Finally, cues to action indicated by American studies included physician’s recommendation and school requirement, and although this was only reported by few studies, a positive association with acceptability was found [14]. In the African studies, cues to action also enclosed endorsement from the government and acknowledgement by community members (associations with acceptability were not investigated) [21]. In general, both reviews showed that the HBM constructs influence people’s willingness to vaccinate against cervical cancer. However, they do caution for overreliance on the results: since almost all studies included were cross-sectional no causal relations could be identified [14, 21].

It is generally agreed upon that there is a need to further test health behavior theories as to justify their use in promotion and vaccination interventions and to verify their applicability in different settings. It is known that the utility of the HBM varies according to the type of behavior that is predicted (preventive versus curative) and the health condition to be tackled (prevalence, morbidity and mortality of the disease in the study setting). Furthermore, cultural or socio-demographic variables might affect the predictive value of the model [19, 24, 25]. According to Janz and Becker, socio-demographic characteristics can have both direct and modifying effects on the (associations between) HBM constructs [19]. With regard to HPV vaccination, characteristics such as cervical cancer knowledge, age of the daughter or conservative thinking often affect acceptability [14, 15]. However, there is no clear description on which are most important and there is no agreement on how such personal characteristics fit the HBM (e.g. directly, mediated, or moderating effects).

Similarly, CTA are poorly studied. In theory, two types are distinguished: internal cues, such as symptoms, and external cues, such as advice from others or a promotional campaign. While these conventional definitions seem straightforward, measuring CTA remains a challenge given that *“a cue can be as fleeting as a sneeze or the barely conscious perception of a poster”* [20]. In addition, to truly be a factor that influences behavior, the trigger does not only have to reach the person, it also needs to prompt adoption of the behavior [26]. So depending on an individual’s perception, a certain cue might be interpreted as a trigger or not. Therefore, we propose to include a personal assessment of a cue such as promotion, expanding CTA to receiving and personally evaluating the motivator, e.g. by using the questions ‘did you receive an invitation for the cervical cancer vaccination program?’ and ‘did you feel well informed?’.

Finally, another point of discussion about the operationalization of the HBM is the outcome measure. While the original HBM had actual behavior as outcome (e.g. ‘vaccine uptake’), many studies apply the HBM to identify factors influencing acceptability or intention, considering these intervening variables as a precursor of behavior [14, 15, 24]. However, attitudes and intentions do not always translate into health behavior [27]. Research should therefore not only include antecedents but also the actual behavior as to distinguish factors that influence willingness versus those that inhibit or drive true behavior. Moreover, theories should be tested through longitudinal studies in which the influence of past behavior – often the biggest predictors of future behavior – is, if possible, excluded [24, 25]. Given that HPV vaccination in Kenya is not yet widespread, a pilot vaccination program offered the opportunity to measure the predictive value of the HBM constructs in this context and to explore the additional value of innovative variables.

The purpose of the present longitudinal study was to examine the applicability of the HBM to predict HPV vaccine uptake in Kenya. This general aim is specified into three underlying research objectives. First, we examined whether the HBM constructs predicted vaccine uptake, including a subjective evaluation of promotion. Second, we evaluated the validity of adding willingness to vaccinate to the HBM as mediator of uptake. Lastly, a hypotheses generating component was added, examining the direct- and modifying effects of personal characteristics on the (associations between the) HBM constructs.

## Methods

### Pilot HPV vaccination program

Through the Gardasil Access Program (GAP), Moi Teaching and Referral Hospital (MTRH – Eldoret) received 9000 doses of the HPV vaccine. Ten out of forty-two public primary schools in Eldoret Municipality were randomly selected to participate in this pilot vaccination program. All girls in classes 4 to 8 of these schools (i.e. around 4000 pupils, approximately 9–13 years old), were eligible to receive three free doses of the quadrivalent vaccine. The vaccination was provided in MTRH, located in the center of Eldoret, while promotion was organized at school: health care providers informed the teachers who then passed on the information to students and parents. Implementation of such promotional activities differed from school to school, from parents meetings at school to teachers asking their pupils to notify their parents about the vaccination opportunity. The baseline and follow-up study took place in March 2012 and May 2013 respectively, i.e. right before and after the pilot program, which ran from May 2012 till March 2013 [28, 29].

### Participants and procedures

**Fig. 1:**
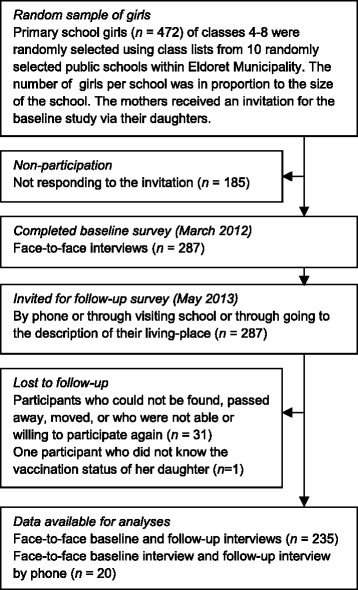
Flow diagram of the recruitment and response of study participants

For this study, a random selection of girls eligible for vaccination were given an invitation letter for the face-to-face baseline interview, addressed to their mother. The number of girls per school was in proportion to the size of the school. Contact information requested at baseline was used to make an appointment for the follow-up interview: participants were contacted by phone, or the interviewers went looking for them at the description of the living-place or at school. If those contacted by phone were not able to participate in the complete, face-to-face follow-up interview, they were invited to answer by phone whether or not their daughter had received the HPV vaccine. (Figure 1) The women were interviewed in Swahili or English, depending on their preference.

During the baseline interview, mothers were given basic information and had the opportunity to ask questions regarding cervical cancer, HPV vaccination and the upcoming program in order to enable them to make an informed decision. More detailed information about the planned vaccination effort was meant to be provided to all parents of all eligible girls by promotional activities at school. To achieve consistency in the interviews, standard guidelines for introductions, interviews, and informed consent requests were practiced [28, 29].

### Measures

#### Outcome variable uptake

The main outcome of the study was the actual behavior, i.e. HPV vaccine uptake, reported by the participants during the follow-up survey (i.e. when the pilot HPV vaccination program had closed). Uptake was reported per dose but assessed as a dichotomous variable (0 = received no HPV vaccine doses, 1 = received one or more doses of the HPV vaccine) given that few vaccinated girls had not completed the required scheme of three doses.

#### HBM constructs

All constructs were measured at baseline (before the pilot HPV vaccination program started). Perceived severity, susceptibility and three barriers (‘foreseeing father’s refusal’, ‘doubting vaccine efficacy’ and ‘perceiving lack of information’) were assessed directly, while other HBM constructs (self-efficacy, trusting the health benefit of the vaccine and the two barriers ‘having safety concerns’ and ‘foreseeing time constraints’) were measured through several items (Table 1). All items were derived from the literature, and benefits and barriers were chosen based on previous research in similar contexts [7, 15, 23, 30, 31].

**Table 1:** Complete list of items used to assess the health belief model (HBM) constructs and willingness

Constructs	baseline/follow-up^a^	Item wording (response options)	# items	α^b^
Severity	baseline	How serious would it be if your daughter would have cervical cancer? (1 = not serious at all–5 = very serious)	1	n/a
Susceptibility	baseline	How likely is it that your daughter would develop cervical cancer in the future? (1 = very unlikely–5 = very likely)	1	n/a
Benefit health	baseline	You would vaccinate your daughter because:	3	.888
		The vaccine will protect her health. (1 = strongly disagree–5 = strongly agree)		
		The vaccine will protect her reproductive health. (1 = strongly disagree–5 = strongly agree)		
		The vaccine will prevent her from having cervical cancer. (1 = strongly disagree–5 = strongly agree)		
Barriers	baseline	You would not vaccinate your daughter because:		
Lack of information		You need more information first (1 = strongly disagree–5 = strongly agree)	1	n/a
Doubt vaccine efficacy		You doubt that the vaccine will truly prevent cervical cancer and genital warts (1 = strongly disagree–5 = strongly agree)	1	n/a
Time constraints		You think vaccination always takes a lot of time. (1 = strongly disagree–5 = strongly agree)	2	.791
		You think it’s inconvenient that she needs 3 doses. (1 = strongly disagree–5 = strongly agree)		
Safety concerns		You think it might have unknown future side effects. (1 = strongly disagree–5 = strongly agree)	3	.882
		You think it might interfere with her fertility. (1 = strongly disagree–5 = strongly agree)		
		You’re afraid the vaccine will not be administered safely (clean needles). (1 = strongly disagree–5 = strongly agree)		
Father’s refusal		You think your partner or her father won’t approve it. (1 = strongly disagree–5 = strongly agree – 0 = no current relationship)	1	n/a
Self-efficacy	baseline	Are you confident that you could let your daughter get vaccinated if you wanted? (1 = not confident at all–5 = very confident)	2	.762
		For you, if you want your daughter to be vaccinated against cervical cancer, that would be. (1 = very difficult–5 = very easy)		
Adequate promotion^c^	follow-up			
Personal level		Did you feel well informed regarding the cervical cancer vaccination program? (0 = no, 1 = yes)	1	n/a
School level		School average of adequate promotion at personal level	1	n/a
Willingness to vaccinate	baseline	Would you vaccinate your daughter against cervical cancer? (1 = very unlikely–5 = very likely)	2	.901
		Will you let you daughter get vaccinated against cervical cancer through this program? (1 = very unlikely–5 = very likely)		

^a^Measure obtained from baseline or follow-up interview

^b^Cronbach’s alpha (α) indicating the reliability

^c^Participants were asked if that had heard about the HPV vaccination program at the hospital after being informed during the baseline interview. If yes, they were asked whether or not they had felt well-informed

#### Mediator willingness to vaccinate

This variable was composed of the sum score of 2 baseline items, i.e. ‘Would you vaccinate your daughter against cervical cancer?’, and, ‘Will you let you daughter get vaccinated against cervical cancer through this program?’ (Table 1).

#### Adequate promotion

During the follow-up interview, people were asked whether they had heard of the HPV vaccination program through school after the baseline interview and if so, whether they felt well-informed regarding the cervical cancer vaccination program. Through this we assessed if promotional activities had reached the women (cfr. CTA) and how the messages were perceived. Adequate promotion was thus a subjective evaluation of outreach messages. Since promotion differed among schools, we created a variable reflecting the level of adequate promotion in each school (i.e. the average of being well-informed at personal level for each school). This variable captured the ‘school effect’, i.e. the different levels of promotion among the different schools, while the original variable measured being well-informed at personal level (Table 1).

#### Personal characteristics

We included ten personal factors and socio-demographic variables to explore their potential direct and modifying effects on the HBM constructs, willingness and uptake: (1) age of the participant; (2) age of the daughter; (3) class of the daughter; (4) marital status of the participant; (5) number of children (<18 years) in the household; (6) ever heard of cervical cancer (awareness participant); (7) years of schooling of the participant; (8) origin of the participant: whether the participant grew up in an urban or rural area; (9) religion of the participant; (10) socio-economic status (SES): a scale representing the quality of the building materials used for the house. All these factors were obtained from the baseline survey [28]. For some of the items (marital status, origin, and religion) answer options in the questionnaire were merged based on preliminary analysis and to facilitate interpretations.

### Statistical analyses

To compare participants who completed versus participants who did not complete the follow-up survey, we performed an univariate analysis of variance (ANOVA). Cronbach’s alpha was calculated to check for internal consistency of constructs’ items (>0.75 was considered acceptable) [32]. Personal characteristics with less than 5 % missing data were imputed using the expectation maximization method (EM), after establishing that the data were missing completely at-random (Little’s MCAR *χ* ^*2*^(259) = 257.583*, p* = .513). If a background characteristic had more than 5 % missing values, only data from participants without missing values was used to build models including that variable. Pathway modeling was applied to investigate the three specific research objectives.

The first research objective, evaluating whether the HBM predicts HPV vaccine uptake in a Kenyan context, was examined with two models: Model 1, containing the HBM constructs measured at baseline (perceived severity, susceptibility, benefits, barriers, and self-efficacy) and Model 2, adding adequate promotion, measured at follow-up, as predictor for uptake.

The second research objective, assessing the validity of adding willingness to vaccinate to the HBM as mediator of uptake, was examined with Model 3. This model contained all the predictors of Model 2. However, the baseline HBM constructs were specified to predict willingness, and willingness and adequate promotion to predict uptake.

Finally, we examined the direct and modifying effects of all ten personal characteristics on the (associations between the) HBM constructs. To do so, we applied an exploratory modeling procedure. First, all factors were independently added as direct (e.g. heard of cervical cancer → uptake), mediated (e.g. religion → severity → willingness), or moderating effect (e.g. age of the daughter → willingness, with barrier ‘father’s refusal’ moderating the effect) in Model 3 (Additional file 1: Table S1). Next, Model 4 was fitted containing all significant effects from this exploratory procedure in addition to the predictors specified in Model 3. To correct for multiple testing we applied a more conservative critical *p*-value of 0.01.

Models were fitted using the weighted least-squares estimator with mean and variance adjustment (WLSMV; because of the dichotomous primary outcome variable (uptake)) [33]. To ensure reliable interpretation of the results, the underlying assumptions of SEM were checked for all variables included in the models (multicollinearity, linearity in the logit, missing data, and outliers). Furthermore, the nine baseline HBM constructs were allowed to correlate in all models. In addition, all models were evaluated by assessing the efficacy of each model in predicting willingness and uptake (*R* ^2^ *)*. Since uptake is a dichotomous variable, *R* ^*2*^ is estimated assuming that the categorical indicator is a coarse categorization of a normally distributed underlying dimension. Furthermore, the fit of the path models (Model 3-Model 4) was assessed with the chi-square overall goodness-of-fit statistic (CHISQ), the comparative fit index (CFI), the Tucker-Lewis Index (TLI), the root-mean-square error of approximation (RMSEA), and the weighted root mean square residual (WRMR). RMSEA values <0.06, CFI >0.97, TLI >0.9 and WMSR < 1.0 indicate close fit [34, 35]. MPlus was used to perform the analyses.

### Sample size

The necessary sample size was calculated for a previous study and data analysis [28]. With 255 observations, the data set is however also adequate for SEM (i.e. minimum 200 observations) [34].

### Ethics, consent and permissions

The Institutional Research and Ethics Committee of Moi Teaching and Referral Hospital, and the Ethical Committee of Ghent University Hospital approved this study (approval numbers FAN:IREC 000771 and B670201212980-B670201317007, respectively). Written informed consent was requested before the baseline interview, and this was verbally confirmed before the follow-up interview. Participants received no incentives for participation in the baseline survey, while a financial compensation of 200 Kenyan Shilling (US $2.34) was given for the time and effort they invested in a second face-to-face interview [28].

## Results

### Participation

A flow diagram of recruitment and response of participants within this longitudinal research design is presented in Fig. 1. Of the 472 invited participants, only 287 agreed to participate in the baseline survey (61 %), while 256 of them (89 %) agreed to participate in the follow-up survey. Non-completers (*n* = 31) were similar to completers (*n* = 256) on all HBM constructs and personal characteristics with only one exception. Compared to completers, the non-completers scored slightly lower on self-efficacy (t(285) = 2.547, *p* = 0.011).

Of the 256 participants of the follow-up survey, 8 % (*n* = 20) only provided the information about their daughter’s vaccination status through the short telephone survey; data on adequate promotion is missing for them. One participant was deleted from analysis because she did not report whether her daughter was vaccinated (*N*_analyses_ = 255).

The baseline HBM constructs did not have any missing values and Cronbach’s alpha was found to be acceptable (>.75) for all HBM constructs (Table 1) [32].

### Descriptive analysis

Of the 255 participants included in the analyses, the average willingness to vaccinate was 4.4 (range 1–5). >This positive attitude towards the HPV vaccine was reflected in the baseline measured HBM constructs: the average perceived severity was 3.8 (range 1–5), average perceived susceptibility was 3.7 (range 1–5), average perceived health benefits was 4.6 (range 1–5). Furthermore, proposed barriers were not often agreed on. The average scores on the barriers (range 1–5) were: lack of information 3.5 (range 1–5), doubting vaccine efficacy 2.4, time constraints 1.4, safety concerns 2.6, and father’s refusal 1.5. Lastly, the average score of mothers’ self-efficacy was 4.3 (range 1–5) (Table 2).

**Table 2:** Correlations, means, standard deviations, and ranges of Health Belief Model constructs

Variable	1	2	3	4	5	6	7	8	9	10	11	12	13
Correlation coefficients:													
1. vaccine uptake	1.00												
2. Willingness to vaccinate	.13*	1.00											
3. Severity	.00	.25*	1.00										
4. Susceptibility	.02	.38*	.21*	1.00									
5. Benefit health	.08	.34*	.31*	.06	1.00								
6. Barrier lack of information	.02	.02	.06	-.09	.22*	1.00							
7. Barrier doubt vaccine efficacy	-.01	-.15*	.03	-.14*	-.07	.38*	1.00						
8. Barrier time constraints	-.03	-.21*	-.18*	-.08	-.29*	-.02	.10	1.00					
9. Barrier safety concerns	.00	-.15*	.03	-.17*	.02	.37*	.79*	.10	1.00				
10. Barrier father’s refusal	-.08	-.39*	-.09	-.28*	-.13*	.09	.26*	.19*	.29*	1.00			
11. Self-efficacy	.18	.54*	.27*	.15*	.56*	.11	-.03	-.32*	-.02	-.32*	1.00		
12. Adequate promotion: individual	.36*	.14*	.03	.05	.04	-.07	.06	-.05	.05	-.17*	.11	1.00	
13. Adequate promotion: school	.36*	.16*	.07	.07	.09	.02	.09	-.07	.10	-.08	.15*	.42*	1.00
Mean^a^	31 %	4.43	4.82	3.79	4.60	3.51	2.37	1.38	2.55	1.54	4.33	62 %	.62
SD	.46	.86	.59	1.03	.58	1.47	1.35	.56	1.29	1.43	.81	.47	.20
Range	0/1	1-5	1-5	1-5	1-5	1-5	1-5	1-4	1-5	0-5	1-5	0/1	.18-.83

*N* = 255

^a^ Means of dichotomous variables are replaced by proportions of ones observed

In the follow-up survey, 37 % of the participants mentioned they had not been well-informed about the program (adequate promotion = 0). However, the average percentage of people mentioning this lack of promotion fluctuated per school (18 % - 83 %). By the end of the program, 31 % had their daughter vaccinated against cervical cancer with one dose or more (72 % of them had received 3 doses). Means, standard deviations, and correlations across HBM constructs, willingness to vaccinate, adequate promotion and vaccine uptake are provided in Table 2.

Of all personal characteristics, four had less than 5 % missing values (age of the daughter, age of the participant, cervical_cancer_awareness and origin_of_the_participant) and one had more than 5 % (years of schooling of the participant). The characteristics of the participants can be summarized as follows: the average age of the mothers was 36 (range 21–59); the average age of the daughter was 12 (range 8–18); the average class of the daughter was 6 (range 4–8); 76 % of the participants had a partner (married or living together) while the remaining 24 % was either separated, widowed or never had a partner (i.e. currently single); 3.5 (range: 1–7) was the average number of children in the household; 60 % had at least heard of cervical cancer (awareness); the average years of schooling of the participant was 8.4 (range: 0–16); 60 % of the participants grew up in a Kenyan city, 38 % were originally from the countryside and 1 % was from outside Kenya (the latter 2 were grouped for analysis); 80 % of the participants indicated to be Protestant, 15 % Catholic, 4 % Muslim, 1 % other or no religion (for analyses this was combined into Muslim (4.3 %) vs. non-Muslim (95.7 %)); and the average score of the quality of the building materials of the house was 4.6 (range 2–7) (Table 3).

**Table 3:** Baseline characteristics of the participants (*n* = 255)

	Mean^a^	Range	Standard deviation
Characteristics of the mother			
Age of the mother	36	21-59	6.8
Mother has a partner	76 %	0-1	0.4
Years of schooling	8.4	0-16	3.5
Raised in a urban area (vs rural)	60 %	0-1	0.5
Cervical cancer awareness at baseline	60 %	0-1	0.5
Islamic (vs. other religion)	4.3	0-1	0.2
Characteristics of the household			
Quality of the house	4.6	2-7	0.9
Number of children	3.5	1-7	1.3
Characteristics of the daughter			
Age of the daughter	12	8-18	2.0
Class of the daughter	6	4-8	1.4

^a^Means of dichotomous variables are replaced by proportions of ones observed

### The health belief model

#### Research objective 1: Application of the HBM

**Fig. 2:**
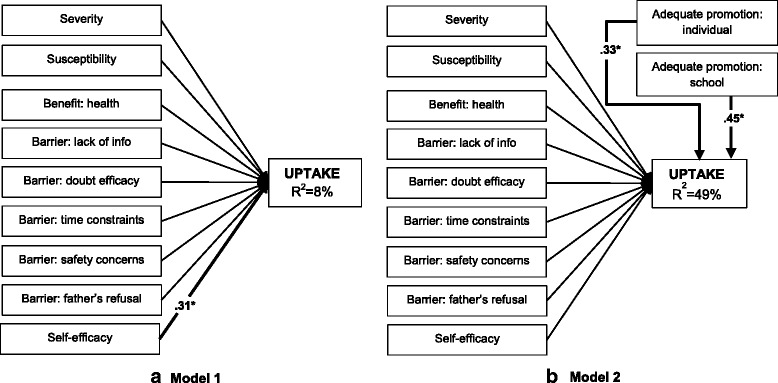
Health Belief Model to predict HPV vaccine uptake without adequate promotion (**a** Model 1) and with adequate promotion (**b** Model 2). Numbers represent the significant (*p* < .05) standardized parameters (*β*). Thin lines without numbers represent non-significant parameters in the model. *R* ^2^ represents the explained variance of the dependent variable. (*N* = 255)

First, to examine how the nine HBM constructs measured at baseline predicted uptake, we fitted Model 1 (Fig. 2a). The nine predictors only accounted for 8 % of the variance in uptake. The only significant predictor of uptake was self-efficacy (standardized path coefficient self-efficacy *β* = .31).

In Model 2 (Fig. 2b) it was examined whether addition of the two adequate promotion variables increased the explained variance of uptake. The two included adequate promotion variables, adequate promotion at the individual level (*β* = .33) and adequate promotion at the school level (*β* = .49), increased the explained variance of uptake to 49 %. None of the other predictors were significantly related to uptake.

#### Research objective 2: Willingness as a predictor for uptake

**Fig. 3:**
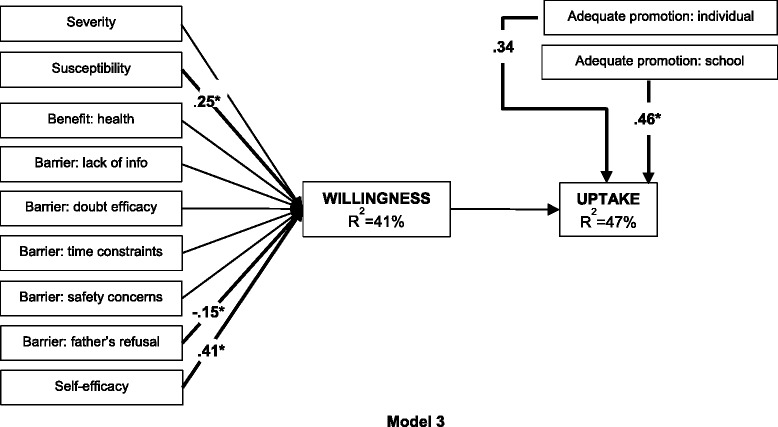
Health Belief Model to predict HPV vaccine uptake fully mediated by willingness to vaccinate [CHISQ(11) = 7.276, *p* = 0.776; RMSEA = 0.00; CFI = 1; TLI = 1.08; WRMR = 0.51]. Health Belief Model to predict HPV vaccine uptake fully mediated by willingness to vaccinate (Model 3). Numbers represent the significant (*p* < .05) standardized parameters (*β*). Thin lines without numbers represent non-significant parameters in the model. *R* ^2^ represents the explained variance of the dependent variable. (*N* = 255)

Model 3 (Fig. 3) assessed the validity of adding willingness to vaccinate to the HBM as mediator of uptake. In Model 3, the nine baseline HBM constructs were specified to predict willingness, and willingness and adequate promotion to predict uptake. Model 3 provided a close fit to the data [CHISQ(11) = 7.276, *p* = .776; RMSEA = .00; CFI = 1; TLI = 1.081; WRMR = 0.51]. Overall, 47 % of the variance in uptake and 41 % of the variance in willingness was explained by predictors in the model. Willingness was not significantly associated to uptake. In contrast, adequate promotion at the individual level (*β* = .34) and at the school level (*β* = .46) were significantly related to uptake. Susceptibility (*β* = .25), the barrier ‘foreseeing father’s refusal’ (*β* = −.15), and self-efficacy (*β* = .41) were significantly related to willingness.

#### Research objective 3: Influence of personal characteristics in the HBM

Finally, in Model 4 we examined the direct and modifying effects of personal characteristics on the (associations between the) HBM constructs. A priori, an exploratory modeling procedure was applied examining the effects (1. direct, 2. mediated, 3. moderating) of each personal parameter individually. Of the direct effects of the characteristics, only cervical cancer awareness was found to be significantly (*p* < .01) related to uptake. Next, the effect of religion (i.e. being Muslim) on willingness was found to be significantly mediated (*p* < .01) by: severity, susceptibility, self-efficacy, ‘trusting the health benefit’, and the barriers ‘foreseeing time constraints’ and ‘foreseeing father’s refusal’. Furthermore, the age of the daughter was found to have a significant (*p* < .01) effect on the relation between barrier ‘foreseeing father’s refusal’ and willingness. Lastly, marital status and SES had a significant (*p* < .01) effect on the relation between susceptibility and willingness (Additional file 1: Table S1).

**Fig. 4:**
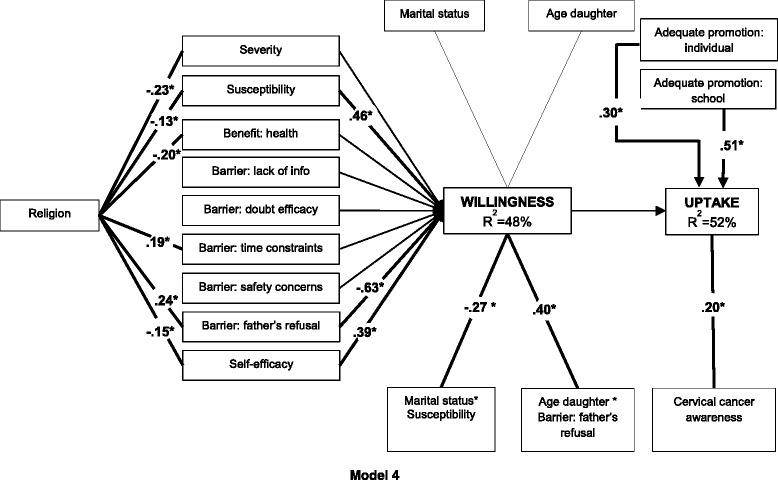
Health Belief Model to predict HPV vaccine uptake including personal characteristics and mediated by willingness [CHISQ (85), *p* = 0.0001, RMSEA = 0.052; CFI = 0.920; TLI = 0.84; WRMR = 0.910]. Health Belief Model to predict HPV vaccine uptake including personal characteristics and fully mediated by willingness to vaccinate (Model 4). Numbers represent the significant (*p* < .05) standardized parameters (*β*). Thin lines without numbers represent non-significant parameters in the model. *R* ^2^ represents the explained variance of the dependent variable. (*N* = 255)

In Model 4 (Fig. 4) we added all these significant effects of the personal characteristics to Model 3. To avoid estimation errors, only the strongest of the interactions with susceptibility (i.e. susceptibility*marital status) was incorporated in the model. The predictors in the model explained 48 % of the variance in willingness and 52 % of the variance in uptake; willingness was not significantly associated with uptake (*p* = 0.185). Religion was found to be significantly (*p* < .05) related to severity (*β* = −.23), susceptibility (*β* = −.13), self-efficacy (*β* = −.15), ‘trusting the health benefit’ (*β* = −.20), and the barriers ‘foreseeing time constraints’ (*β* = .19), and ‘foreseeing father’s refusal’ (*β* = .24). Next, susceptibility, the barrier ‘foreseeing father’s refusal’, and self-efficacy were related to willingness (*β* = .46, *β* = −.63, *β* = .39 respectively). Furthermore, two interactions were significantly related to willingness: marital status*susceptibility (*β* = −.27), and age of the daughter*barrier ‘foreseeing father’s refusal’ (*β* = .40). Lastly, in addition to adequate promotion at individual level (*β* = .30) and at school level (*β* = .51), baseline cervical cancer awareness was significantly related to uptake (*β* = .20). Acceptable goodness of fit was obtained with Model 4 [CHISQ (85), *p* = 0.0001, RMSEA = .052; CFI = 0.920; TLI = 0.836. WRMR = 0.910].

## Discussion

The Health Belief Model is an established health theory often used as framework to develop health interventions. In this model, constructs concerning severity, susceptibility, benefits, barriers and self-efficacy are considered important determinants of the health related behavior [17–20]. This study examined whether the HBM can be applied to predict HPV vaccine uptake in Kenya, a country with little research on HPV vaccine acceptability and uptake.

### Research objective 1: Application of the HBM, including adequate promotion

A first remarkable result of this study was the large difference between Model 1 and Model 2: adding adequate promotion, at both personal and school level, increased the predictive value from 8 to 49 %. The strong correlation between adequate promotion and HPV vaccination is not surprising since many studies have stressed the importance of triggers such as health provider’s recommendation [16, 36–38]. Our results might, however, overestimate the strength of the association because of two reasons: 1) Unlike the other HBM constructs, adequate promotion is measured at follow-up, i.e. when uptake was also recorded, which means the direction of the correlation is indeterminable, and 2) adequate promotion reflects the quality of the promotion from the perspective of the participant. This means that two participants who received the same information through the same channel, might report adequate promotion differently, most possibly in agreement with the vaccination status of their daughter. Nevertheless, the strong correlation cannot be overlooked: whether or not the daughter received the vaccine was highly associated with obtaining sufficient information. Furthermore, it is important to mention that before adequate promotion was added to the model, self-efficacy was the only HBM construct found to have a positive correlation with vaccine uptake. This clearly shows that besides an external trigger, participants still need to perceive themselves capable in performing the action, i.e. taking their daughter for a vaccination, and therefore justifies addition of this construct to the HBM.

The fact that none of the other HBM constructs predicted uptake is surprising, yet there are several explanations possible. First of all, threat (severity and susceptibility) and ‘trusting the health benefit’ are very skewed, making it more difficult to identify relations. All participants considered cervical cancer as a very severe disease which their daughter was (very) likely to get, and they all were driven to protect their daughter’s health. Given that cancer is perceived severe and deathly worldwide, it is a not a startling ascertainment that also in Kenya, where treatment remains inaccessible for many people, cervical cancer is considered a serious disease. Moreover, severity has often been identified as a construct with less predictive value, definitely with regards to preventive behavior [19, 39–41]. With regard to susceptibility, one can wonder how well parents are capable to estimate future (sexual) behavior and well-being of their daughter. Do they overestimate their daughter’s vulnerability because of concern and anxiety? Such emotions clearly also influence decision-making yet they are not included in cognitive theories [20, 42]. Finally, the current HIV epidemic, affecting all layers of society, might have increased their sense of vulnerability regarding sexual transmittable infections.

Barriers are very often among the strongest predictors of behavior [19, 40], but in our study none were associated with uptake. Again, little variance was found: almost all participants trusted the efficacy and safety of the vaccine and worried little about time boundaries or objection of their partner. Social desirability and poor assessment skills of the participants might be at the base of these highly pro-vaccine statements. On the other hand, other studies found similar results and the worldwide success of childhood vaccination might also encourage Kenyan women to truly trust and welcome the new HPV vaccine, as other studies have also found [7–9]. Future studies can explore this more in-depth e.g. by applying more multiple item measures, since they have better predicting power, or by assessing users’ and non-users’ perspectives during and after program implementation. While this latter approach would not contribute to identifying causal relations it could help to explore and identify other determinants than the HBM constructs given that in this study we found little or no support for the HBM in the current context of cervical cancer vaccination in Kenya.

### Research objective 2: Willingness as a predictor for uptake

Adding willingness to vaccinate as mediator of uptake lowered the predictive value of the HBM from 49 to 47 %. Moreover, willingness had no effect on vaccine uptake, while adequate promotion remains highly associated. These results raise the issue of control, i.e. to what extent are people truly in control of vaccination behavior if they are depending on providers’ motivation and initiation? As stated by Sheeran P. (2011), the gap between intention and behavior is caused by those with high intention who don’t act (inclined abstainers) and those with low intentions who do act (disinclined actors) [27]. In the case of this HPV vaccination pilot program, it seems that many participants are inclined abstainers as a result of poor promotion, i.e. they wished to vaccinate their daughter against cervical cancer but were not well enough informed to do so. On the other hand, we need to ask ourselves the question how well people can express their wish and predict their behavior in this context. Again, socially desirable answers may have caused overestimation of willingness, but there are many other *factors* [27] that may have led to expression of high interest and/or low uptake. Most participants had never heard of the HPV vaccine and 40 % had never heard of cervical cancer. For them to process all information received during the baseline interview and immediately report acceptability and intention to vaccinate might have been difficult or unreliable (*cognitive variables*) [27, 42]. In addition, the time-lapse between the first interview and the start of the pilot program, might have given participants time to overthink (*temporal stability*) and discuss cervical cancer vaccination with friends and family (*subjective norms*). As a result, some participants might have changed their opinion and preferred not to act [27, 37, 43]. Finally, other important activities (*competing intentions*) might have inhibited participants from taking the time to let their daughter get vaccinated against cervical cancer [27]. Given the harsh living circumstances of many of our participants, other priorities are not unlikely.

The nine baseline HBM constructs, which only explained 8 % of the variance of uptake (Model 1), explained 41 % of the variance of willingness. Given that willingness to vaccinate was also measured at baseline (as opposed to uptake at follow-up), it was expected to detect more correlations among the cross-sectional data. Self-efficacy was the strongest correlate, but also susceptibility was positively associated. Perceived vulnerability has been previously related with acceptability [10, 36] and uptake of (preventive) behavior [16, 18–20], yet as described above, we did not find the latter correlation. Finally, participants who thought of their partner as somebody who would oppose to vaccinate their daughter against cervical cancer, were less likely to accept the vaccine. Interventions should target these characteristics and include all decision makers as to increase the willingness to vaccinate.

### Research objective 3: Influence of personal characteristics in the HBM

Personal characteristics altered Model 3 and increased the explained variance of willingness from 41 to 48 % and of uptake from 47 to 52 %. However, given that only acceptable goodness of fit was achieved, we merely consider this as a sketch on how these variables are related with HBM constructs, willingness and uptake as opposed to an adapted version of the model. For example, awareness had a direct impact on uptake which supports the importance of cognitive variables: participants who had heard of cervical cancer before baseline were more likely to vaccinate their daughter. Whether the effect is a result of knowledge of cervical cancer rather than the ability to process the new information regarding the vaccine more easily, is yet to be determined. Also, religion clearly affected the HBM constructs: Muslims were more likely to agree with the barriers ‘father’s refusal’ and ‘time constraints’, were less likely to perceive cervical cancer as severe, thought their daughter was less susceptible, had lower self-efficacy, and were less driven by the fact that the vaccine would protect their daughter’s health. The underlying reasons, e.g. a more conservative attitude or mistrust in the health system, are to be investigated more in-depth. Finally, the positive effect of susceptibility on willingness was higher for single mothers, and the negative relation of perceiving the father as a barrier for willingness weakened when the daughter was older. While the former interaction might reveal a kind of freedom to express intentions among women without a partner, the latter hints that even though a partner may object, mothers of older girls still intended to vaccinate, maybe without his consent. Our results suggest that personal characteristics influence vaccination differently in different circumstances, demonstrating the complexity of the decision-making process regarding cervical cancer vaccination. Further research is necessary to define whether or not some of these variables would have an added value to the HBM.

## Conclusions

We found little support for the HBM in the context of HPV vaccination in Kenya and neither was willingness a good predictor for uptake. During the past few years, the term vaccine hesitancy has popped up in vaccination literature regarding reluctance towards immunization, referring to “*to delay in acceptance or refusal of vaccination despite availability of vaccination services*”. Measuring vaccine hesitancy, and its determinants vaccine confidence, complacency and convenience, might offer a better insight in the ‘state of preparedness’ and willingness of people to vaccinate against cervical cancer as opposed to acceptability or intention to vaccinate, which are now mostly used in formative research [44].

However, other longitudinal studies have equally showed that attitudes, health beliefs and intentions are not always strong correlates of HPV vaccination [37, 38, 45]. Reiter et al. proclaim that *“beliefs and attitudes may not be important determinants in the early adoption of behaviors that are not well understood by most individuals”* [37]. In the same light and based on the strong correlation between adequate promotion and vaccine uptake, we hypothesize that supportive important others, motivation by health providers and general trust in the health system may be of extreme importance to counteract knowledge gaps and doubts. Therefore, we recommend to further study whether interpersonal variables and variables at the level of community or health system are (more) important determinants of new (preventive) health actions as opposed to personal beliefs [42, 46]. By monitoring future HPV vaccination programs and by assessing users’ and non-users’ perspectives these variables could be more explored and if deemed appropriate added to the HBM. Furthermore, such research could help identifying specific components of promotion interventions necessary for the target group to perceive promotion as adequate. Finally, our results also encourage the examination of modifying effects of personal characteristics since they might boost the predictive value of the HBM. Identification of such determinants might then help to increase the efficacy of future promotion campaigns and as such, create awareness, consensus and support for HPV vaccination at the community level.

## Additional file

Additional file 1: Table S1. Direct, mediated, and moderating effects of socio-demographic variables (X_sdv_) on Health Belief Model constructs (X_hbmc_), willingness and vaccine uptake: unstandardized path coefficients (β). (DOCX 21 kb)
