# Supplementary material for: Uptake of the human papillomavirus vaccine in Kenya: testing the health belief model through pathway modeling on cohort data
Source: Global Health. 2016 Nov 15;12:72. doi: 10.1186/s12992-016-0211-7 (PMC5111174; doi:10.1186/s12992-016-0211-7)
Supplement: Additional file 1: Table S1. — Direct, mediated, and moderating effects of socio-demographic variables (Xsdv) on Health Belief Model constructs (Xhbmc), willingness and vaccine uptake: unstandardized path coefficients (β). (DOCX 21 kb) [file 12992_2016_211_MOESM1_ESM.docx]

**Additional file 1**

| **Table S1.** Direct, mediated, and moderating effects of socio-demographic variables (x_sdv_) on Health Belief Model constructs (x_hbmc_), willingness and vaccine uptake: unstandardized path coefficients (β). | | | | | | |
| --- | --- | --- | --- | --- | --- | --- |
|  | **Socio-demographic variable (x_sdv_)** | **N*^a^*** | **Direct effect of x_sdv_ on Uptake^b^** | **By x_HBMc_ mediated effect of x_sdv_^c^** | **Moderating effect of x_sdv_ on the relations in Model 3^d^** | **Direct effects of interaction terms^e^** |
|  | Age of participant | 255 | -- | -- | -- | -- |
|  | Age of daughter | 255 | -- | -- | Age daughter * Barrier father’s refusal- Willingness | Age daughter –Willingness - |
|  |  |  |  |  | 0.049 (0.015)* | 0.047 (0.039)* |
|  |  |  |  |  |  | Barrier father’s refusal -0.689 (0.179)* |
|  | Class of daughter | 255 | -- | -- | -- | -- |
|  | Marital_status of participant (single vs. | 255 | -- | -- | Marital_status * Susceptibility-Willingness | Marital_status-Willingness |
|  | with partner) |  |  |  | -0.267 (0.086)* | 1.543 (0.321)* Susceptibility- |
|  |  |  |  |  |  | Willingness 0.385 (0.072)* |
|  | Number of children in household | 255 | -- | -- | -- | -- |
|  | Heard of cervical cancer | 255 | 0.664 (0.213)* | -- | -- | -- |
|  | Schooling of participant | 236 | -- | -- | -- | -- |
|  | Origin of participant (urban vs. rural) | 255 | -- | -- | -- | -- |
|  | Religion of participant (non- | 255 | -- | Severity -0.661 (0.086)* | -- | -- |
|  | Muslim vs. Muslim) |  |  | Susceptibility -0.628 (0.239)* |  |  |
|  |  |  |  | Benefit health -0.558 (0.113)* |  |  |
|  |  |  |  | Barrier time constraints 0.505 (0.104)* |  |  |
|  |  |  |  | Barrier father’s refusal 1.036 (0.275)* Self-efficacy |  |  |
|  |  |  |  | -0.523 (0.153)* |  |  |
|  | Socio-economic status (SES) **^f^** | 255 | -- | -- | SES*Susceptibility-Willingness-0.116 (0.038)* | SES-Willingness 0.419 (0.130)* Susceptibility- |
|  |  |  |  |  |  | Willingness |
|  |  |  |  |  |  | 0.716 (0.157)* |
| *^*^p* <.01; -- = indicating that no significant (direct/moderated/mediating) effects were found for the socio-demographic variable; standard errors are in parentheses.  ^a^ If a background characteristics had more than 5% missing values, models, verifying the direct, mediated of moderated effect of the variable, were compared using only data from participants who had no missing values.  ^b^ Direct effects were examined through specifying a direct effect of the personal characteristic on vaccine uptake within Model 3.  ^c^ Mediated effects were examined through specifying direct effects from the personal characteristic to the ten predictors in Model 3.  ^d^ Moderating effects on the relations in Model 4 were examined for all personal characteristics separately .  ^e^ Direct effects of the variables in the moderation on dependent x_HBMc_.  **^f^** A score representing the quality of the building materials of the house. | | | | | | |
